# Deep Learning-Based Instance Segmentation of Galloping High-Speed Railway Overhead Contact System Conductors in Video Images

**DOI:** 10.3390/s25154714

**Published:** 2025-07-30

**Authors:** Xiaotong Yao, Huayu Yuan, Shanpeng Zhao, Wei Tian, Dongzhao Han, Xiaoping Li, Feng Wang, Sihua Wang

**Affiliations:** 1 School of Electronic & Information Engineering, Lanzhou Jiaotong University, Lanzhou 730070, China; yxt@lzjtu.edu.cn (X.Y.); crystalciky@outlook.com (H.Y.); 2 School of Automatic & Electrical Engineering, Lanzhou Jiaotong University, Lanzhou 730070, China; 3 China Railway Beijing Group Co., Ltd., Beijing 100038, China

**Keywords:** high-speed railway OCS, deep learning, conductor galloping monitoring, YOLO11, instance segmentation

## Abstract

The conductors of high-speed railway OCSs (Overhead Contact Systems) are susceptible to conductor galloping due to the impact of natural elements such as strong winds, rain, and snow, resulting in conductor fatigue damage and significantly compromising train operational safety. Consequently, monitoring the galloping status of conductors is crucial, and instance segmentation techniques, by delineating the pixel-level contours of each conductor, can significantly aid in the identification and study of galloping phenomena. This work expands upon the YOLO11-seg model and introduces an instance segmentation approach for galloping video and image sensor data of OCS conductors. The algorithm, designed for the stripe-like distribution of OCS conductors in the data, employs four-direction Sobel filters to extract edge features in horizontal, vertical, and diagonal orientations. These features are subsequently integrated with the original convolutional branch to form the FDSE (Four Direction Sobel Enhancement) module. It integrates the ECA (Efficient Channel Attention) mechanism for the adaptive augmentation of conductor characteristics and utilizes the FL (Focal Loss) function to mitigate the class-imbalance issue between positive and negative samples, hence enhancing the model’s sensitivity to conductors. Consequently, segmentation outcomes from neighboring frames are utilized, and mask-difference analysis is performed to autonomously detect conductor galloping locations, emphasizing their contours for the clear depiction of galloping characteristics. Experimental results demonstrate that the enhanced YOLO11-seg model achieves 85.38% precision, 77.30% recall, 84.25% AP@0.5, 81.14% F1-score, and a real-time processing speed of 44.78 FPS. When combined with the galloping visualization module, it can issue real-time alerts of conductor galloping anomalies, providing robust technical support for railway OCS safety monitoring.

## 1. Introduction

Railways are a nation’s crucial infrastructure and transportation network, whose safety and stability are directly related to national security, people’s livelihood, and economic development. As of September 2024, China’s high-speed railway operating mileage reached 46,000 km, accounting for nearly two-thirds of the world’s total high-speed rail network [1,2]. With the continuous expansion and upgrading of the high-speed railway network and the sustained growth in passenger and freight traffic, ever higher demands are placed on the reliability of the OCS (Overhead Contact System).

The contact suspension in the railway OCS directly interfaces with the pantograph of electric locomotives to enable power transmission. Moreover, several auxiliary cables, including positive feeders and protective wires, are essential for boosting the electromagnetic environment of the OCS, augmenting power supply capacity, and maintaining the safety of power transmission. Under harsh weather conditions such as strong winds, sandstorms, and snow or ice, OCS conductors are highly susceptible to conductor galloping, which can lead to wear of the wires and fittings, inter-wire arcing, and wire breakage, posing serious threats to train operation safety and the stability of the power supply. Due to the abrupt and variable characteristics of conductor galloping, conventional manual inspection techniques are ineffective, labor-intensive, and considerably constrained in harsh environmental conditions. Therefore, there is an urgent need for automated monitoring systems to replace or supplement manual inspections and improve safety management and control [3].

Current methods for monitoring conductor galloping can be categorized into two primary types [4]: the first relies on traditional image-processing edge-detection algorithms, which delineate conductor contours by calculating grayscale gradients using operators such as Sobel and Canny; however, these methods are prone to significant noise interference in complex backgrounds, during occlusion, or when conductors deform, failing to reconcile fine details with overarching semantics [5]. The second type employs deep-learning feature extraction and instance segmentation techniques, utilizing end-to-end networks to autonomously learn the nonlinear characteristics of conductors across various orientations and deformations, producing pixel-level masks that accurately reconstruct conductor structures while mitigating background noise [6]. At the same time, deep networks can meet high-frame-rate real-time inference requirements, thereby enabling online, automated conductor galloping monitoring.

Traditional approaches have primarily focused on image enhancement and classical edge detection. Yu et al. [7] improved the Canny algorithm by using the first derivative of the Cauchy distribution as the edge detection function, thereby enhancing its performance; Soria et al. [8] proposed the TEED algorithm, which constructs a lightweight yet efficient edge detection model that achieves strong generalization and real-time performance across multiple datasets; and Tian et al. [9] introduced a weighted nuclear norm minimization-based Sobel edge detection method, performing image denoising prior to edge extraction to improve robustness in high-noise environments. Moreover, Chen et al. [10] presented an adaptive-threshold Sobel edge detection method based on an improved genetic algorithm, automatically computing the optimal threshold in place of manual setting, and Akshi Kumar et al. [11] enhanced image edges via guided image filtering and applied an improved ant colony optimization algorithm to the filtered image for edge detection.

To fulfill the criteria for real-time performance and stability, some studies have explored image-processing-based approaches. Li Xin et al. [12] proposed a line-segment segmentation and detection method for infrared overhead high-voltage line images, using an improved Hough line detection algorithm combined with a bimodal histogram method and image differentiation to extract conductor segments, achieving progress in image line-segment segmentation; Li Baokun et al. [13] constructed a semantic segmentation dataset for transmission-line conductors, streamlined the HED network architecture, and transformed the transmission-line detection problem into a pixel-level classification task, providing a solution for pixel-level classification of transmission-line conductors; and Zeng Yaoyun et al. [14] addressed the insufficient detection efficiency and accuracy of live-work robots on transmission lines under complex lighting and background conditions by proposing a multimodal feature-extraction method that combines edge detection based on an improved Canny operator with Hough-transform line detection, achieving enhanced detection speed in complex scenarios.

To address the demands for online monitoring and small-object detection, another line of research focuses on multimodal data and edge-computing platforms. To overcome the challenge of reliably extracting power lines against complex backgrounds, Tiago Santos et al. [15] proposed a UAV-based power-line detection method using multi-modal deep learning; by compensating for the field-of-view differences between the two cameras, their approach achieves accurate wire segmentation. Long Shanshan et al. [16] addressed low efficiency in transmission-line defect detection by clustering multi-view images with the K-means algorithm, enabling automated recognition of transmission-line defects. Tian Yunlong et al. [17] tackled insufficient accuracy in anomalous defect detection on transmission lines by introducing an edge-computing-based multi-scale convolutional neural network; their method extracts multi-scale features of anomalies to localize and classify transmission-line defects. Zhao Jielun et al. [18] presented a transmission-line defect detection method based on a scale-invariant feature pyramid, enhancing small-object defect detection accuracy through multi-scale feature fusion.

Some studies have investigated detection speed improvements to enhance model efficiency in resource-constrained environments. Salehi et al. [19] proposed a deep learning network for automatic railway detection and segmentation from aerial imagery, employing a convolutional network and fully connected layers to form a lightweight classifier for detection and segmentation; Liu Yanmei et al. [20] addressed the slow speed of existing transmission-line detection methods by replacing the backbone with MobileNetV2 and introducing attention mechanisms and a void pyramid pooling method to realize the lightweight of transmission line detection; Liu Yepeng et al. [21] proposed a multi-scale object detection method for transmission lines based on the FP-SSD algorithm, replacing the traditional VGG network with ResNet and integrating feature pyramid structures for information fusion across feature layers, demonstrating strong adaptability in transmission-line object-detection scenarios; and Yanbin Weng et al. [22] tackled low accuracy and long inference time in UAV aerial images by proposing a DA-DeepLabv3+ algorithm, replacing the original Xception backbone with MobileNetV2 and integrating Dense-ASPP and multi-scale attention mechanisms to balance accuracy and speed.

At the same time, to enhance the network’s response to critical structures, many studies have integrated attention modules or high-order feature fusion. Chen Youkun et al. [23] systematically compared the performance differences in VGG16, MobileNetV2, and DenseNet as backbone networks, providing valuable insights for addressing segmentation challenges in interwoven transmission-tower-line scenarios; Shihan Liu et al. [24] proposed an efficient, low-complexity, anchor-free detector based on an improved YOLO, designed a new stochastic loss function, and introduced a lightweight decoupled head, achieving significant improvements in inference speed and small-object detection performance while maintaining accuracy; and Yang Cheng et al. [25] proposed a PL-UNeXt model that adds an edge-detail head and a line-feature head to assist feature extraction, optimized for the complex backgrounds of aerial images.

In conclusion, despite the excellent outcomes achieved by traditional methodologies in the segmentation and detection of slender linear targets, challenges remain, including the misidentification of conductor edges and the inefficiency of current models. To address these issues, we propose an enhanced YOLO11-seg model, integrating a Four-Direction Sobel Enhancement (FDSE) module for multi-directional edge extraction, an Efficient Channel Attention (ECA) mechanism to adjust channel weights, and Focal Loss to prioritize challenging samples for improved instance segmentation of OCS conductors. This model enables real-time monitoring of conductor galloping by analyzing mask differences between contiguous frames, facilitating early detection and mitigating economic losses caused by natural disasters, while ensuring the uninterrupted functioning of railway OCS systems. The objective of this research is to improve detection accuracy, efficiency, and real-time monitoring capabilities for more reliable railway OCS systems.

## 2. Materials and Methods

### 2.1. OCS Conductor Dataset

The OCS conductor dataset was independently constructed by the research team and covers realistic conditions from multiple railway lines across China. The data were collected using smartphones (Samsung S23 + smartphone, Samsung Electronics Co., Ltd., Suwon, Gyeonggi-do, South Korea; Xiaomi 14 Pro smartphone, Xiaomi Communications Co., Ltd., Beijing, China), UAVs(DJI Mavic 3, SZ DJI Technology Co., Ltd., Shenzhen, Guangdong, China; DJI Air 3S, SZ DJI Technology Co., Ltd., Shenzhen, Guangdong, China), and fixed surveillance cameras (SHL-019 industrial camera, Shenzhen Shunhuali Electronics Co., Ltd., Shenzhen, Guangdong, China), ensuring diversity in both temporal and environmental aspects. Specifically, the dataset includes conductor galloping captured at different times of day such as early morning, midday, and evening, under various weather conditions such as sunny, cloudy, rainy, foggy, and snowy days, and across multiple conductor states such as mild sustained oscillation, low-frequency large-amplitude swing, and severe galloping.

**Figure 1:**
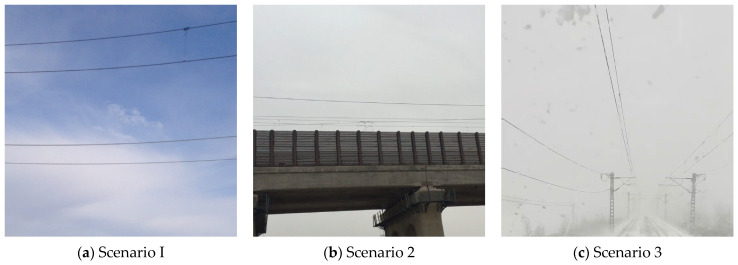
Conductors galloping under different scenario.

Although only three representative images are shown in this paper, the actual dataset encompasses a wide range of complex and realistic operating conditions. Figure 1 displays selected representative samples that highlight variations across time, weather, and conductor motion states. This dataset provides sufficient experimental data and diverse scenario support for subsequent tasks such as instance segmentation and the safety monitoring of OCS conductors.

### 2.2. Dataset Processing

Considering the high annotation cost, redundant frames, and limited training resources associated with video data, only static images were used as the data source during model training; after training, the model could be applied to both images and video frames at inference. Images with clearly visible conductor states were selected, and key frames showing clear conductor states were extracted from the collected videos, yielding over 900 original images. To enhance the model’s ability to recognize targets under different environmental conditions, the extracted images underwent data augmentation—rotation, scaling, color jitter, random cropping, and the addition of various types of noise—expanding the dataset to 1275 images [26]. The dataset was then split into an 8:1:1 ratio, resulting in 1020 training images, 128 validation images, and 127 test images. These preprocessing steps provided a rich and diverse sample set to support subsequent model training.

### 2.3. Dataset Annotation

**Figure 2:**
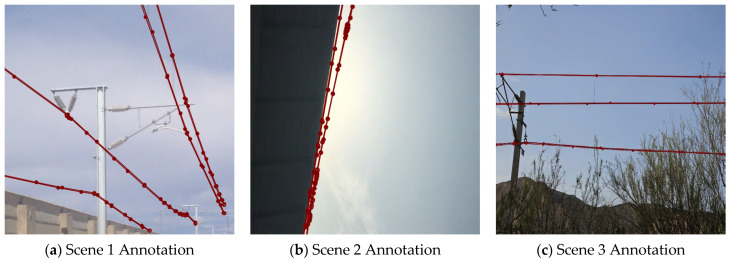
Dataset annotation results.

Using LabelMe, images were annotated for segmentation by precisely marking OCS conductor contours to generate polygon annotation data. Subsequently, custom conversion scripts were used to transform the JSON-format annotation files into the TXT format required by YOLO, which contains the object class and normalized bounding-box coordinates. The annotation results under different weather conditions and scenarios are shown in Figure 2.

### 2.4. Overall Instance Segmentation Model Design

YOLO11-seg is a lightweight, efficient instance segmentation model upgraded from YOLOv8-seg. Through multiple structural and training strategy optimizations, it significantly reduces model complexity while improving segmentation accuracy. First, YOLO11 introduces more efficient CSP modules and feature fusion mechanisms in both the backbone and neck networks, coupled with an improved data augmentation scheme, reducing the parameter count of the nano-scale model by approximately 22% compared to YOLOv8-nano. Second, the segmentation head design was upgraded from an anchor-based architecture to an anchor-free, pixel-level mask-prediction pipeline, greatly enhancing mask-generation precision and inference speed. Finally, the default integration of Distribution Focal Loss in YOLO11, through fine modeling of the discrete distribution in bounding-box regression, strengthens small-object localization and bounding-box fitting capabilities, thereby boosting overall detection and segmentation performance.

The choice of YOLO11-seg for this task stems from its ability to effectively balance high detection accuracy with real-time performance. YOLO11-seg, an upgraded version of YOLOv8-seg, provides an excellent balance of speed and accuracy, making it well-suited for real-time applications like galloping monitoring of high-speed railway OCS conductors. Furthermore, YOLO11-seg’s lightweight architecture, with efficient CSP modules and feature fusion mechanisms, allows it to process complex edge details and small-object detection efficiently, which are crucial for the accurate monitoring of dynamic conductor behavior. The model’s anchor-free design, which eliminates the need for pre-defined anchor boxes, also contributes to its flexibility and better handling of various object scales. These features, combined with its robust performance in dynamic environments, make YOLO11-seg an ideal choice for this application.

In the network backbone, the input image first undergoes two standard convolution operations. This is followed by a 256-channel FDSE module, which introduces four-direction Sobel edge enhancement to fuse edge information from the input feature map with deep convolutions. The hyperparameter *α* is introduced within the FDSE module to dynamically adjust the fusion weight between the edge-enhanced feature branch and the residual feature branch. After experimental validation, the optimal value of *α* = 0.4 was found, striking the best balance between edge detection accuracy and overall segmentation performance. Multiple rounds of standard convolution–FDSE operations then further strengthen the model’s perception of dynamic conductor details. Finally, an SPPF module aggregates the feature maps, and a C2PSA module reorganizes inter-channel information to provide rich, multi-scale features for subsequent detection and segmentation.

In the network’s Neck and Head portions, ECA modules are integrated at the multi-scale feature-fusion nodes in layers 14 and 18 to adaptively weight channel features, allowing the model to automatically enhance conductor-related critical information while diminishing redundancy. We tested multiple sets of hyperparameters for the ECA module, and after experimentation, the optimal values of *γ* = 1.8 and *b* = 1 were found to yield the best results on our contact-wire conductor dataset. The ECA module is lightweight and comprises minimal parameters, so its application to essential layers with merely 512 and 256 channels has a negligible effect on the total inference speed of the network.

**Figure 3:**
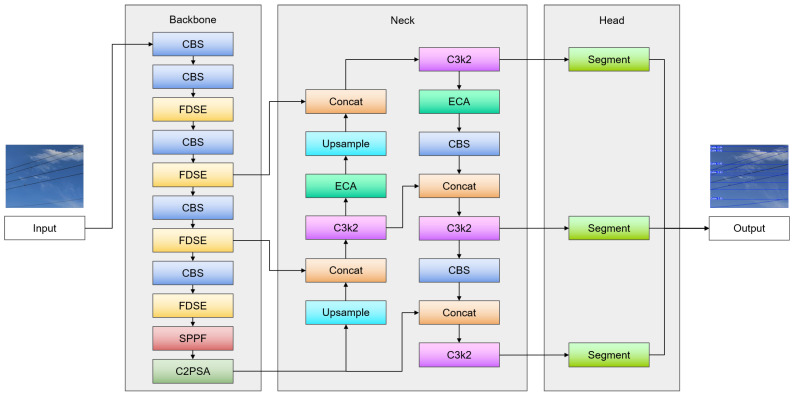
Schematic of the improved YOLO11-seg.

The usual cross-entropy loss is ultimately substituted with Focal Loss, with hyperparameters configured to *α* = 0.25 and *γ* = 1.5 following experimental validation. Focal Loss incorporates a modulating factor that diminishes the weight of well-classified samples, prompting the model to concentrate on difficult-to-classify instances during training, which subsequently enhances overall detection and segmentation performance, as well as the model’s accuracy and robustness. Figure 3 illustrates the design of the enhanced network model.

### 2.5. Sobel Edge Detection Enhancement

Sobel edge extraction is a traditional picture edge detection technique that calculates image intensity gradients and their directional variations through convolution operations to delineate edge structures. This approach excels in images characterized by significant intensity gradients and elevated noise levels, providing precise edge localization and effectively detecting edge variations. The Sobel operator enhances transition areas between conductors and the backdrop, consequently augmenting the efficiency and precision of conductor galloping identification and segmentation.

The conventional Sobel operator consists of two 3 × 3 kernels for calculating first-order gradients in the horizontal (X) and vertical (Y) orientations, respectively. The magnitudes of these directional gradients are subsequently amalgamated by a weighted sum to augment edge responses, as seen in Equations (1) and (2).

(1) $$G_{x}=\sum_{i=-1}^{1}\sum_{j=-1}^{1}K_{x}(i,j)\cdot I(x+i,y+j)$$

(2) $$G_{y}=\sum_{i=-1}^{1}\sum_{j=-1}^{1}K_{y}(i,j)\cdot I(x+i,y+j)$$

**Figure 4:**
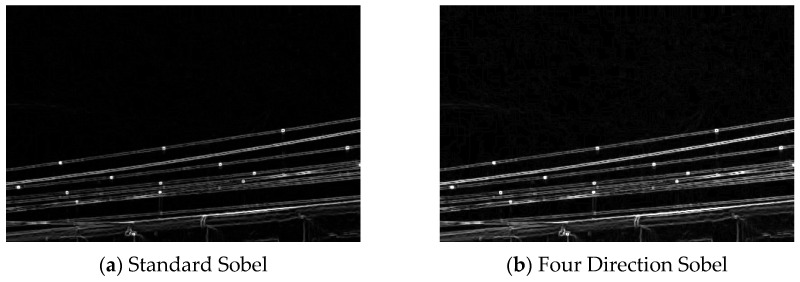
Comparison of standard Sobel and four-direction Sobel edge detection.

This operator is inadequate for extracting diagonal features and fails to capture higher-order edge information in complex scenes, as it relies predominantly on horizontal and vertical detection and only captures first-order gradient changes, thereby restricting its utility in advanced vision tasks. In practical situations, high-speed railway OCS conductors can gallop in many directions, displaying curved, twisted, or intersecting edge formations in photographs. A four-direction Sobel filter is presented, which incorporates 45° and 135° diagonal edge-detection kernels. By utilizing four 3 × 3 filter matrices aligned with the four primary orientations (0°, 45°, 90°, and 135°), edge features are extracted in all these directions to address the shortcomings of the conventional Sobel operator and improve diagonal feature extraction. Figure 4 illustrates a comparison of the edge-extraction efficacy of the conventional Sobel operator and the four-direction Sobel filter.

### 2.6. FDSE Module

The original YOLO11 model utilizes a Cross Stage Partial (CSP) architecture, wherein the input image undergoes convolution, batch normalization, and activation functions to generate a foundational feature map, which is subsequently divided into two branches: the primary convolutional branch and a residual branch. The primary branch collects profound features with sequential convolutional layers and bottleneck architectures, whereas the residual branch employs basic convolutions to retain superficial features. The two branches are amalgamated by a Concat process to produce the final feature map.

In intricate situations—such as indistinct edge details and structural deformations resulting from conductor galloping—the basic model’s capacity to detect fine edges is constrained. To address this issue, a four-direction Sobel edge detection technique is integrated into the YOLO11 backbone, introducing the FDSE (four-direction Sobel enhancement) module, which leverages the strengths of both traditional and deep learning algorithms to enhance the model’s ability to extract galloping information.

The core idea of the FDSE module is to send the input feature map in parallel through two branches and fuse their outputs using a hyperparameter *α*, which assigns different weights to each path. The FDSE module integrates four-direction Sobel edge enhancement into the primary branch and utilizes deeper convolutional layers to extract local details and high-level semantic information, thus addressing the limitations of standard convolutions in detecting small edge variations.

To strengthen the network’s capability in capturing slender edge information of conductors, the original C3k2 module at the down-sampling layer of the YOLO11 backbone is replaced by the FDSE module. This modification enhances the extraction of conductor features and improves segmentation accuracy. Following several convolutional stages, the features from the augmented branch are fused with those from the residual branch using a weighted Concat process governed by hyperparameter *α*, assigning weight *α* to the Sobel-enhanced branch and weight 1 − *α* to the residual branch, resulting in a feature map that balances edge sensitivity and semantic depth. The formula is shown in Equation (3).

(3) $$F_{Fusion}=\alpha F_{Sobel}+(1-\alpha )F_{Conv}$$

**Figure 5:**
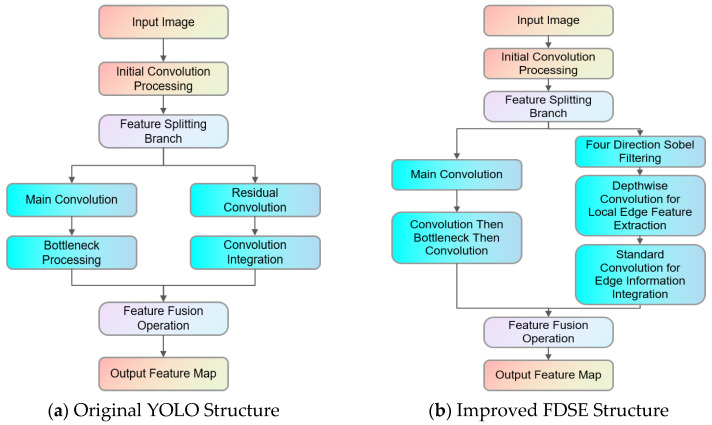
The original YOLO structure and the improved FDSE structure.

Figure 5 illustrates a comparison between the original YOLO11 architecture and the enhanced FDSE structure.

### 2.7. ECA Module

ECA (Efficient Channel Attention) is a lightweight channel attention module whose primary aim is to capture dependencies between feature channels using a one-dimensional convolution without adding excessive parameters or computational overhead, thereby enhancing the model’s channel attention representation capability. Compared with traditional attention mechanisms, ECA omits fully connected layers and dimensionality reduction operations, avoiding potential information loss introduced by attention modules [27].

**Figure 6:**
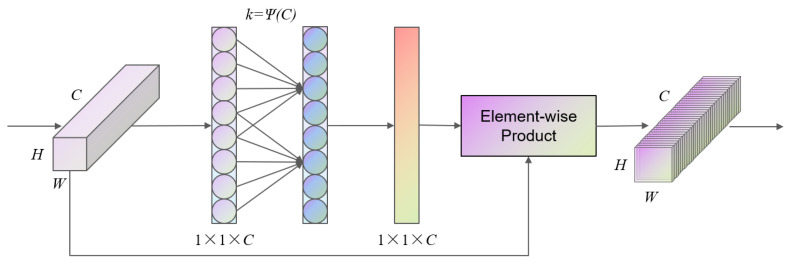
Diagram of the ECA module.

The ECA module applies global average pooling to the input feature map to obtain aggregated features. It then performs a 1 × 1 convolution of size k—where k is adaptively determined by mapping from the channel dimension C—to compute a weight coefficient for each channel. These weights are normalized via a Sigmoid function and applied to the original feature map to enhance important channels and suppress less informative ones. The use of the ECA module is motivated by the fact that details of the contact-wire conductors typically only appear prominently in a few channels, and ECA can adaptively allocate attention weights to each channel, thereby preserving detailed information more effectively. The ECA module is shown in Figure 6.

Moreover, in the YOLO11 block there exists a multi-scale feature fusion layer that not only contains high-resolution information but also integrates low-resolution information, so we insert the ECA module after these two fusion layers; this allows the network to dynamically assign channel weights based on the fused composite features, further improving model performance. By avoiding channel compression and expansion, ECA better preserves edge details and channel-specific features. Moreover, ECA has a low parameter count and computational cost, offering stronger local response capability and lower computational complexity, thus satisfying requirements for both real-time performance and accuracy.

### 2.8. Focal Loss Function

The Focal Loss function is designed to address the class imbalance between positive and negative samples during training, which is a critical factor affecting model performance. In this work, OCS conductors typically occupy only a small portion of the image, while the majority of the image area is background, so most candidate-region pixels are negative samples. This overwhelming number of background pixels relative to target pixels results in a very low proportion of positive samples in the overall data, exacerbating the class imbalance problem [28]. The imbalance between positive and negative samples can render the standard cross-entropy loss insufficient to guide the model to adequately learn the features of hard-to-detect samples. The prediction probability for the true class and the definition of the sample weight are given in Equation (4).

(4) $$p_{t}=\left\{\begin{array}{cc} p, & y=1\\ 1-p, & y=0 \end{array}\right.\;w=\left\{\begin{array}{cc} \alpha , & y=1\\ 1-\alpha , & y=0 \end{array}\right.$$

In the equation, *Pt* is the confidence for label *y*; *p* is the confidence of the sample being predicted as the positive class; *y* is the ground-truth label (*y* = 1 for positive samples, *y* = 0 for negative samples); *ω* is the sample weight; and *α* is the class balance factor.

The traditional cross-entropy loss (CE) minimizes the divergence between the model’s predicted probability distribution and the true distribution, guiding model learning. However, when faced with a large number of easily classified negative samples, CE can cause the model to neglect hard samples, limiting detection accuracy. To mitigate this, YOLO11 adopts the balanced cross-entropy loss (BCE), which assigns different weights to positive and negative samples and includes a weighting factor so that the loss remains effective under class imbalance. Although BCE addresses the imbalance, it cannot distinguish between easy and hard samples. Therefore, Focal Loss is employed. Building on CE and BCE, Focal Loss introduces a dynamically scaled modulating factor that down-weights well-classified (easy) samples during training, quickly focusing the loss on hard-to-classify samples. The Focal Loss is defined in Equation (5); when *γ* = 0, it reduces to the original CE.

(5) $$FL(p_{t})=-w{\left(1-p_{t}\right)}^{\gamma }\log(p_{t})$$

In the equation, *FL*(*Pt*) denotes the predicted confidence for *y*; *γ* is the focusing parameter.

### 2.9. Experimental Environment and Parameter Configuration

All experiments were conducted on a Windows 11 operating system. The software environment comprised Python 3.9.21, PyTorch 2.4.1, and CUDA 12.4. On the hardware side, the experimental platform was equipped with an AMD 7500-f processor, 32 GB of RAM, and an NVIDIA GeForce RTX 4070Ti Super 16 GB GPU. The training parameters for the model are listed in Table 1.

### 2.10. Instance Segmentation Evaluation Metrics

To comprehensively evaluate the performance of the improved instance segmentation model, the following metrics are utilized: *P* (precision), *R* (recall), *AP* (average precision), *F*1 score, and *FPS* (frames per second). *P* represents the proportion of predicted samples that are actually correct, as shown in Equation (6); *R* is the proportion of actual positive samples correctly detected by the model, as shown in Equation (7); *AP* refers to the area under the precision-and-recall curve, as shown in Equation (8). Since this task involves only a single class (conductors), the values of *mAP*@0.5, *mAP*@0.5:0.95, and *AP* are identical, so *AP*@0.5 is used as the average precision metric. *F*1 score is the harmonic mean of *P* and *R*, providing a balanced measure of detection accuracy as shown in Equation (9). *FPS* indicates the number of frames the model can process per second and is a key metric for real-time detection tasks.

(6) $$P=\frac{T_{P}}{T_{P}+F_{P}}\times 100\%$$

(7) $$R=\frac{T_{P}}{T_{P}+F_{N}}\times 100\%$$

(8) $$AP=\int_{0}^{1}P(R)dR$$

(9) $$F1=\frac{2T_{P}}{2T_{P}+F_{N}+F_{P}}=\frac{2\cdot (P\cdot R)}{P+R}$$

In the equation, *TP* (True Positives) is the number of actual positive samples correctly predicted as positive by the model; *FP* (False Positives) is the number of negative samples incorrectly predicted as positive; *FN* (False Negatives) is the number of positive samples incorrectly predicted as negative.

## 3. Results

### 3.1. Comparison with Other Instance Segmentation Models

To evaluate the performance of the YOLO11-seg model and its enhanced version with the FDSE module in object segmentation tasks, we compared it with two mainstream instance segmentation models, Mask R-CNN [29] and DETR [30]. All models were trained using the self-built dataset and training configuration. We compared the performance of these models across multiple metrics, including precision *P*, recall *R*, *AP*, *F*1, and frames per second *FPS*. The experimental results are summarized in Table 2, showing the performance differences among YOLO11-seg, YOLO11-seg with FDSE module, Mask R-CNN, and DETR.

As shown in the table, both Mask R-CNN and DETR have a large number of parameters, which results in high computational resource demands. When applied to detecting contact network conductors, both methods exhibit relatively low accuracy, leading to a higher likelihood of missed detections. Their inference speeds are also slower, with Mask R-CNN achieving only 4.90 *FPS* and DETR reaching 29.47 *FPS*, both of which are lower than YOLOv8’s 41.71 *FPS* and YOLO11’s 33.75 *FPS*, making them unsuitable for real-time applications.

In contrast, YOLOv8 and YOLO11 offer higher accuracy and better recall rates, demonstrating superior overall performance. YOLO11, with fewer parameters, maintains high inference speed, making it suitable for deployment on resource-constrained edge devices. Its inference speed increases from 33.75 *FPS* to 45.29 *FPS*, a gain of 11.54 *FPS*, demonstrating both superior accuracy and real-time performance. These advantages make YOLO11 a more effective solution for real-time object detection tasks.

The improved model not only achieves gains in key metrics such as precision and recall, but also slightly reduces the model’s parameter count, thereby improving detection accuracy. Its overall performance significantly surpasses the original YOLO11 architecture, effectively enhancing the model’s ability to extract and represent features of elongated targets.

### 3.2. Attention Mechanism Improvement Comparison

Because the ECA module was inserted in the Neck, the impact of different attention mechanisms on instance segmentation performance was comprehensively evaluated through the embedding of several other modules—SE [31], SimAM [32], CBAM [33], and DoubleAttention [34]—into exactly the same feature-fusion nodes of the original YOLO11 model. This setup ensures a fair comparison of each module’s effect on model performance. The comparative results are shown in Table 3.

The results show that the ECA module achieves the best overall performance, slightly outperforming the other attention modules on all metrics, with minimal impact on FPS. By maintaining a very low parameter count and computational overhead, it most effectively improves the model’s detection precision and recall.

### 3.3. Loss Function Improvement Comparison

Several common loss functions were compared—YOLO11’s original BCE With Logits [35], Classification Loss, Regression Loss [36], Distribution Focal Loss, and Focal Loss—by integrating each into the model and evaluating their impact on performance, the results are shown in Table 4.

### 3.4. Ablation Study

To verify the contribution of each improvement module to model performance, five controlled experiments were conducted based on the original YOLO11-seg model by adding the FDSE module, ECA module, and Focal Loss, respectively. All ablation experiments were performed under the same hardware, environment, and parameter settings. The results are presented in Table 5.

**Figure 7:**
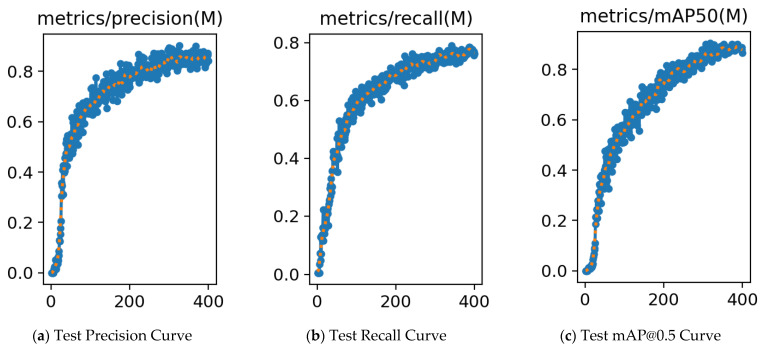
Diagram of the changes in loss and accuracy over iterations.

From the table, it is evident that introducing FDSE, ECA, or Focal Loss individually improves performance to varying degrees. When FDSE, ECA, and Focal Loss are combined with YOLO11-seg’s original efficient multi-scale feature-fusion mechanism, efficient and precise detection and segmentation of OCS conductors is achieved while maintaining real-time inference capability, fully validating the effectiveness of multi-module collaborative gains. Figure 7 illustrates the changes in loss and accuracy over the course of the training iterations of the improved model.

### 3.5. Segmentation Results Comparison

**Figure 8:**
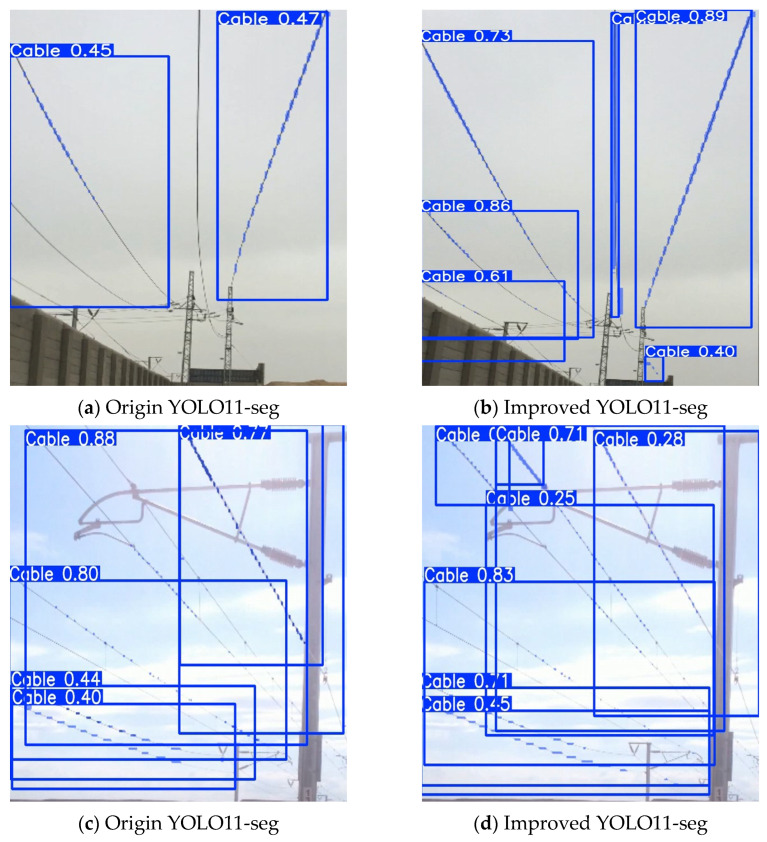
Comparison between the original YOLO11-seg and the improved YOLO11-seg.

Under different weather conditions, segmentation performance is greatly affected by background and lighting. Instance segmentation was performed on galloping OCS conductor images using both the original YOLO11-seg and the improved model to verify the performance gains. Figure 8a,c show the segmentation results of the original YOLO11-seg, while Figure 8b,d show the segmentation results of the improved model.

As Figure 8 illustrates, the original model misses both vertical and diagonal conductors under various weather conditions, produces low confidence scores, and yields unclear mask boundaries. In contrast, the improved model not only more completely captures vertical and diagonal conductor features but also achieves higher confidence and produces noticeably finer segmentation boundaries.

### 3.6. Galloping Determination of OCS Conductors

#### 3.6.1. Mask Extraction

Based on the improved instance segmentation model, the input video is processed frame by frame to obtain pixel-level instance masks of OCS conductors. Leveraging the model’s enhanced ability to detect stripe-like conductor features, accurate contours are extracted even in complex scenes.

**Figure 9:**
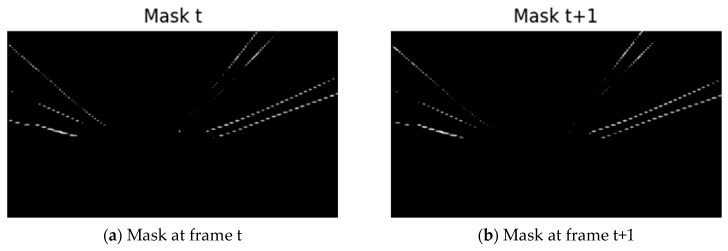
Comparison of mask results.

The masks of conductors in frame t and frame *t* + 1 are binarized separately; pixels with predicted values greater than 0.5 are regarded as conductor targets and assigned a value of 1, and pixels with values less than or equal to 0.5 are regarded as background and assigned a value of 0. The binarized masks are then multiplied by 255 to obtain two standard black-and-white mask images for subsequent calculations. The binarization results are shown in Figure 9a,b.

#### 3.6.2. Mask Differencing and Change Rate Calculation

The two binarized black-and-white mask images are subjected to absolute differencing to count the number of changed pixels. Let *N* be the total number of pixels in each mask. The change rate Δ is defined as the ratio of the number of changed pixels to *N*. This change rate serves as the criterion for galloping detection; if Δ exceeds a predefined threshold, the two adjacent frames are deemed to exhibit conductor galloping. In scenes with strong wind or high conductor stress, the mask difference regions between consecutive frames are large, indicating pronounced galloping; in static or micro-motion states, the difference regions are small or near zero. The computation is given in Equation(10).

(10) $$\Delta =\frac{n}{N}\times 100\%$$

In the equation, *n* is the number of changed pixels; *N* is the total number of pixels in both masks; and Δ is the change rate.

#### 3.6.3. Motion Detection Visualization Results

**Figure 10:**
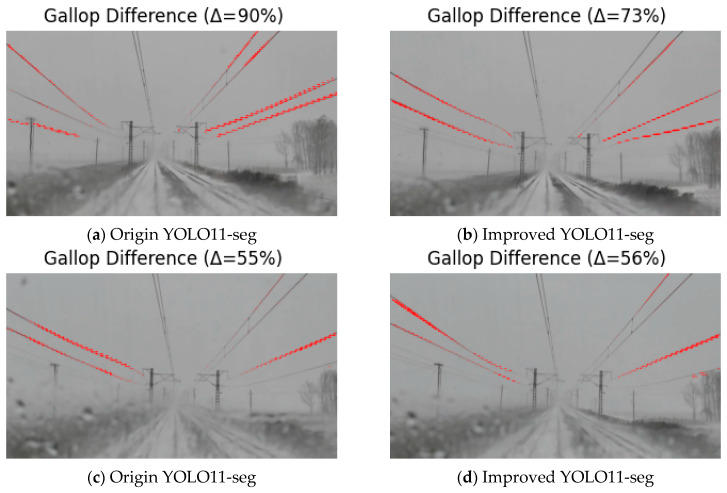
Galloping contour extraction and highlighting.

To more intuitively illustrate the positional changes and motion amplitude of OCS conductors during conductor galloping, target conductors with a small inter-frame mask variation rate Δ (defined in Equation (5) in Section 3.3. Mask Differencing and Change-Rate Calculation) are treated as non-galloping and not displayed, whereas regions with a large Δ undergo contour extraction and are overlaid in red on the original image to visualize the galloping areas. Through this method, conductor galloping can be observed—especially under high wind speeds and intense structural vibrations, when the contour boundaries of the galloping regions become more pronounced and visually salient. An example of the extracted contours of OCS conductors during actual galloping is shown in Figure 10.

By applying efficient image-processing operations to the segmented masks, this method enables OCS conductor galloping detection without the need for additional network structures, validating the effectiveness of the improved model and laying the foundation for subsequent research in trajectory modeling and motion amplitude measurement. The mask-differencing method is simple, computationally lightweight, and interpretable, with adjustable sensitivity by customizing the change rate *Δ* and its threshold, meeting practical needs without the complexity of post-processing algorithms.

## 4. Discussion

### 4.1. Interpretation of Results

#### 4.1.1. FDSE Feature Fusion

The addition of the FDSE module yields the largest single jump in precision and *AP*. By explicitly injecting four-direction Sobel edge features into early and mid-level backbone layers, FDSE strengthens the network’s ability to localize the thin, high-contrast edges of galloping conductors. By processing features in parallel through a Sobel-enhanced branch and a standard convolutional branch, and fusing them with hyperparameter *α*, FDSE flexibly balances edge sensitivity and semantic context. This targeted enhancement reduces boundary uncertainty and leads to a marked reduction in false positives along wire contours, which directly drives the observed improvements in both precision *P* and *AP*.

#### 4.1.2. ECA

When ECA is added on top of FDSE, recall sees its most significant boost. ECA’s lightweight channel-wise attention adaptively amplifies those feature maps most relevant to conductor details while suppressing noisy or background channels. This selective reweighting helps the network recover difficult-to-detect wire segments that might otherwise be lost, improving recall and contributing further to *AP* gains.

#### 4.1.3. Focal Loss

Replacing BCE With Logits Loss with Focal Loss delivers a modest but meaningful increase in *F*1 score. By down-weighting the abundant easy negatives and focusing the learning process on hard positives, Focal Loss alleviates class imbalance and sharpens the decision boundary. This effect is most notable in the boost to the *F*1 metric, indicating a more balanced trade-off between precision and recall.

### 4.2. Comparison with Existing Research

Our approach demonstrates clear advantages over existing methods in several key aspects. First, it excels in accurately detecting fine details, especially under challenging conditions such as conductor galloping, where edges are often blurred or indistinct. Unlike many traditional models, which struggle to maintain edge sharpness in these scenarios, our method ensures precise localization of conductor edges, even in complex environments. Additionally, our model achieves an excellent balance between real-time performance and computational efficiency, allowing for high detection accuracy without sacrificing processing speed. This makes our method ideal for deployment in real-time systems, where performance must be maintained at high frame rates without excessive computational costs. Furthermore, it shows exceptional robustness under varying environmental conditions, such as low-light or strong backlighting, where many existing approaches fail to perform reliably. Despite these challenges, our method maintains stable performance with minimal fluctuations in precision and recall. Moreover, our method addresses the common issue of class imbalance, typically seen in conductor detection, by focusing more on rare and challenging conductor pixels, ensuring that these small but critical features are detected accurately. This leads to significant improvements in both detection and segmentation. Lastly, the overall efficiency of the model, in terms of computational resources and parameter size, is optimized, making it feasible for deployment on resource-constrained devices without compromising accuracy or performance. In sum, our method outperforms existing solutions by providing a more accurate, efficient, and adaptable solution for conductor galloping detection.

### 4.3. Robustness and Applicability

To validate the model’s stability under extreme conditions, the training data were augmented with targeted samples representing low-light, strong back-lighting, stormy weather, and camera vibration scenarios. Experimental results show that under these challenging conditions, the model’s precision *P* decreases only from 83.56% to 78.51%, recall *R* decreases from 75.95% to 71.48%, average precision *AP* decreases from 83.56% to 79.70% and *FPS* drops from 44.78 to 40.29, indicating limited performance fluctuation. The model maintains high segmentation accuracy and real-time inference capability despite noise interference and illumination changes. However, when illumination is completely absent or extremely low, optical images cannot capture valid conductor contours, causing the segmentation task to fail. The results are presented in Table 6.

## 5. Conclusions

The study proposes an improved YOLO11-seg instance segmentation model for high-speed railway OCS conductor galloping detection, addressing key challenges such as edge blurring and dynamic conductor behavior under complex environmental conditions. The integration of the FDSE module and ECA mechanism, combined with the use of Focal Loss, significantly enhances model accuracy and robustness.

The improved model demonstrated substantial improvements in precision, recall, and average precision, as well as a notable increase in *FPS*, making it more suitable for real-time applications. Additionally, the novel approach of using adjacent-frame mask differencing for galloping detection, coupled with intuitive motion visualization, provides an efficient and interpretable solution for monitoring conductor motion.

However, despite the improvements, several limitations remain for the practical application of the proposed model in real-world monitoring systems. The performance of the model heavily depends on the resolution of optical imaging sensors, as lower resolutions may result in poor segmentation accuracy. Moreover, the model faces challenges in extreme low-light or strong backlighting conditions, where edge extraction and motion detection could lead to misdetections. Background interference from features resembling conductor width and texture, such as utility pole shadows or branches, may also cause false positives.

Future work could focus on improving the model’s robustness in complex lighting conditions and addressing issues related to background interference. Additionally, exploring the integration of multi-modal sensors and enhancing the model’s scalability for real-time deployment in embedded systems will be crucial for further advancing OCS conductor monitoring technology.

## Figures and Tables

**Table 1:** Model training parameters.

Parameter	Value
Optimizer	SGD
Learning rate	0.01
Epochs	400
Batch size	64
Momentum	0.937
Weight decay	0.0005

**Table 2:** Comparison with other instance segmentation models.

Module	*P*/%	*R*%	*AP*%	*Parameters*	*F*1/%	*FPS*
Mask R-CNN	57.13	53.85	56.37	41,767,592	55.49	4.90
DETR	60.81	65.39	66.11	41,336,672	63.02	32.47
YOLOv8	70.10	68.71	69.24	3,263,811	69.21	41.71
YOLO11	73.54	67.98	74.41	2,842,803	70.65	33.75
FDSE	79.76	72.25	79.87	2,439,395	75.82	45.29

**Table 3:** Performance comparison of different attention mechanisms.

Module	*P*/%	*R*%	*AP*%	*Parameters*	*F*1/%	*FPS*
SE	75.07	69.12	75.81	40,960	71.97	31.67
SimAM	74.85	68.34	75.03	0	71.45	33.19
CBAM	76.18	70.08	76.21	41,058	73.00	30.36
DAttention	75.91	69.39	75.48	245,760	72.50	28.70
ECA	76.25	70.32	76.36	10	73.17	33.14

**Table 4:** Performance comparison of different loss functions.

Module	*P*/%	*R*%	*AP*%	*F*1/%
BCE With Logits	75.82	69.78	75.39	72.67
Classification Loss	76.29	70.03	76.33	73.03
Regression Loss	75.31	68.96	75.12	72.00
Complete IoU Loss	76.17	70.85	75.71	73.41
Focal Loss	76.42	70.69	76.83	73.44

**Table 5:** Ablation study results on the self-constructed dataset.

No.	FDSE Module	ECA Module	Focal Loss	*P*/%	*R*%	*AP*%	*Parameters*	*F*1/%	*FPS*
1	× *	×	×	73.54	67.98	74.41	2,842,803	70.65	33.75
2	√	×	×	79.76	72.25	79.87	2,439,395	75.82	45.29
3	×	√	×	76.25	70.32	76.36	2,842,813	73.17	33.14
4	×	×	√	76.42	70.69	76.83	2,842,803	73.44	33.75
5	√	√	×	82.47	74.61	81.82	2,439,405	78.34	44.79
6	√	×	√	82.65	75.98	82.29	2,439,405	79.17	44.78
7	×	√	√	79.14	73.03	78.77	2,842,813	75.96	33.16
8	√	√	√	85.38	77.30	84.25	2,439,405	81.14	44.78

* Note: × indicates the module was not used; √ indicates it was used.

**Table 6:** Performance under complex conditions.

Scene	*P*/%	*R*%	*AP*%	*F*1/%	*FPS*
Sunny	83.56	75.95	83.56	79.63	44.78
Low Light	78.51	71.48	79.70	74.82	40.29
Strong Back-lighting	80.06	73.23	81.82	76.55	42.49
Stormy Weather	79.68	73.47	80.79	76.38	41.71
Simulated Vibration	80.89	74.09	82.10	77.42	42.97

## Data Availability

Data are contained within the article.
